# The radio-induced immune response: ballistics is key

**DOI:** 10.1186/s12967-023-04692-5

**Published:** 2023-11-15

**Authors:** Céline Mirjolet, Jérémy Baude

**Affiliations:** 1 Department of Radiation Oncology, Centre Georges-François Leclerc, Dijon, France; 2 Preclinical Radiation Therapy and Radiobiology Unit, Centre Georges-François Leclerc, Dijon, France; 3 UMR INSERM 1231, TIReCS Team, Dijon, France

Dear Editor,

Radiation therapy (RT) is now known to be a powerful immunomodulatory agent. Tumor irradiation leads to the release of double-stranded DNA, sensed via the cGAS/STING pathway [1]. This triggers the production of IFN-I which mainly recruits dendritic cells and improves their antigen presentation, promoting a CD8^+^ tumor-infiltrating lymphocyte (TIL)-dependant antitumor immune response. The release of tumor neoantigens and danger-associated molecular patterns (DAMPs) also participates in making the TME more immunogenic. RT can also play immunosuppressive roles. For instance, it can aggravate hypoxia and ROS synthesis in tumors and stimulate the secretion of immunosuppressive cytokines. Overall, this results in the inhibition of immune effector cells and the accumulation of immunosuppressive cells such as Treg, MDSC and TAM2, both monocyte-derived populations [2]. The balance between these immunoactivating and immunosuppressive effects depends on multiple variables such as RT dose, fractionation [3]. Most of this understanding is based on preclinical studies using a RT techniques (referred to as shield RT (SRT) [4]) which spares most of the animal during irradiation but cannot avoid the exposure of healthy tissues located in the irradiation field. However, RT has considerably improved in the past 20 years and modern RT now allows the accurate deliverance of high doses to the tumor while dramatically limiting the irradiation of healthy tissues (referred to as conformal RT (CRT)). Whether the radio-induced immune response found after SRT also occurs after CRT remains unclear. Therefore, the objective of Tadepalli et al. was to study the intratumoral radio-induced immune response after CRT (Fig. 1) [4].

**Fig. 1:**
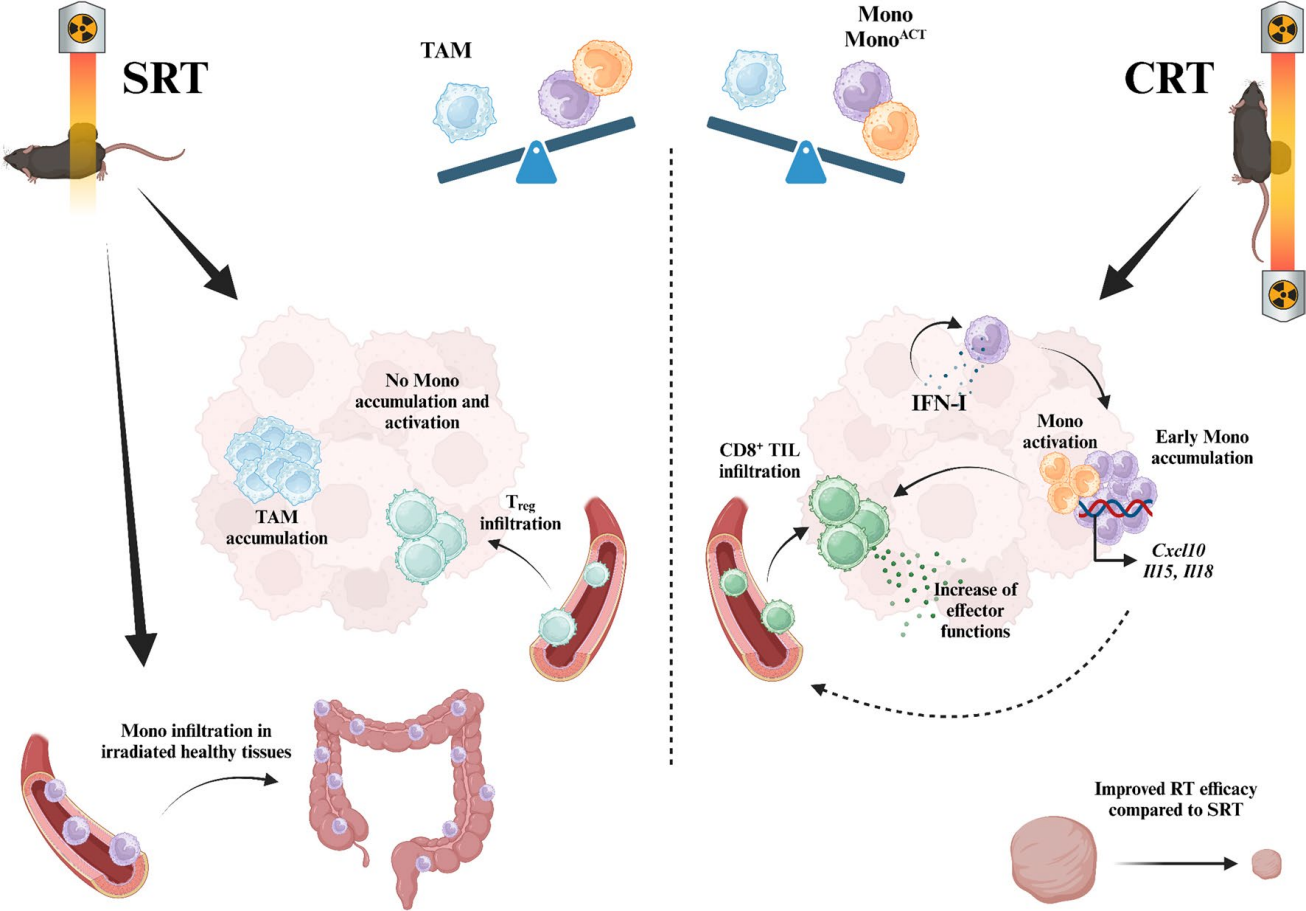
Ballistics and exposure of healthy tissues to radiation impact the radio-induced immune response and RT efficacy. *SRT* shield radiotherapy, *TAM* tumor-associated macrophage, *Mono* monocyte, *Mono*^*ACT*^ activated monocytes, *CRT* conformal radiotherapy, *T*_*reg*_ regulatory T cell, *IFN-I* type I interferon, *TIL* tumor-infiltrating lymphocyte, *Cxcl10* c-x-c motif chemokine ligand 10, *Il* interleukin, *RT* radiotherapy

They first showed that CRT was more effective than SRT at delaying tumor growth and improved survival in a dose and fractionation independent manner due to a more effective acute CD8^+^ TIL-mediated immune response. CRT was responsible for an accumulation of CD8^+^ TILs in the tumor whereas SRT mainly triggered an ingress of T_reg_. Moreover, infiltrated CD8 + T cells had enhanced effector functions after CRT but not after SRT.

To explain these differences which occur even though the same dose was delivered through both RT modalities, the authors pinpointed that CRT elicited a greater early recruitment of monocytes in the TME than SRT. Moreover, whereas monocytes tended to differentiate into TAMs after SRT, they mostly acquired an “activated monocyte” (mono^ACT^) phenotype after CTR, characterized by lower levels of Ly6C and CD115 and higher levels of major histocompatibility complex II (MHC II), CD86 and PD-L1. Surprisingly, this monocyte and mono^ACT^ infiltration resulted in a monocyte-derived cGAS/STING-independent IFN-I secretion promoting monocyte accumulation and monocyte activation again following a positive feedback loop. These populations were therefore critical for activating polyfunctional CD8^+^ TILs after CRT. Finally, normal tissues received significant dose after SRT (but not CRT), resulting in an increased gut permeability, gastrointestinal damage and the relocation of monocytes into the colon whilst not causing lymphopenia which might have accounted for the differences between the two types of RT. This suggests that discrepancies between CRT and SRT probably lie in the relocation of monocytes to healthy tissues after they receive a significant dose.

These results demonstrate that the RT technique and ballistics not only affect the toxicity linked to unintentional healthy tissue irradiation, but also extensively impact the radio-induced immune response and eventually RT efficacy. This underlines the importance of more accurately describing the irradiation technique used in forthcoming preclinical studies: although RT dose, fractionation and the type of particles used are often found in the methods section, the precise ballistics and dose-volume histograms for organs at risk are often missing although they probably largely impact the outcomes as highlighted here. Tumor volume (TV) also appears to be key in studying the radio-induced immune response. Indeed, TVs in the present study were rather small when RT started (i.e. 50–100 mm^3^) and the immune TME described might be different from that obtained after the irradiation of much larger tumors.

This work also paves the way to a new understanding of immune mechanisms underlying RT efficacy. ‘Classical’ pathways largely described to explain the radio-induced immune response, such as the cGAS/STING pathway, albeit important, may not account for the majority of radio-induced effects. Beyond the interest of the improvement of knowledge on the matter, this aspect is paramount, as promising results from the combination of STING agonists and RT found with SRT in preclinical studies might be disillusioning once in the clinic [5].

Altogether, Tadepalli et al. bring a brand new antigen-independent and dendritic cell-independent model to the polyfunctional CD8^+^ TIL-related efficacy of conformal RT through the accumulation and activation of monocytes and their secretion of IFN-I. This was not found when healthy tissues were irradiated, demonstrating that the exposure of organs at risk has a negative therapeutic effect on the antitumor response.

## Data Availability

Not applicable.
